# Global research on Chinese martial arts (1974–2025): A bibliometric and visualization-based analysis using Web of Science

**DOI:** 10.1097/MD.0000000000043769

**Published:** 2025-08-08

**Authors:** Wei Chen, Syahrul Ridhwan Morazuki

**Affiliations:** a Faculty of Educational Sciences and Technology, Universiti Teknologi Malaysia, Skudai, Johor, Malaysia; b Physical Education Department, Shaanxi Railway Institute, Weinan, People’s Republic of China.

**Keywords:** bibliometric analysis, Chinese martial arts, international collaboration, scientific development, Tai Chi

## Abstract

**Background:**

Chinese martial arts (Wushu), representing both competitive sports and cultural heritage, have gained global recognition for their health-promoting benefits. However, a systematic scientometric overview of global Wushu research is still lacking.

**Objective:**

This study systematically examines global research trends, thematic hotspots, collaboration networks, and scientific impacts of Chinese martial arts from 1974 to 2025.

Materials and Methods: This study systematically retrieved English-language articles and reviews from the Web of Science Core Collection (SCI-Expanded and SSCI) published between January 1974 and March 2025, using topic-related terms such as “Wushu,” “Chinese Martial Arts,” “Kung Fu,” “Taijiquan,” and “Tai Chi.” A total of 3955 records were included. The search strategy was refined through multiple rounds of preliminary retrieval and keyword calibration to ensure data accuracy and reproducibility. Bibliometric and scientometric analyses were conducted using Bibliometrix (R version 4.4.3) and its Biblioshiny interface, VOSviewer (version 1.6.20), and CiteSpace (version 6.2.6). The analysis covered publication trends, keyword co-occurrence, collaboration networks, thematic evolution, and citation structures. In addition, linear regression modeling was employed to assess the goodness of fit for annual publication trends.

**Results:**

Global Wushu research has expanded significantly since the 2000s, with an annual growth rate of 9.41% and a peak of 373 articles in 2022. Research prominently focuses on Tai Chi for balance enhancement, fall prevention, chronic disease management, and mental health, especially in older adults. Keywords such as “Tai Chi,” “exercise,” and “older adults” dominate. China (41.9%) and the United States (24.4%) lead in publications; however, China’s international collaboration (20.6%) is lower compared to the US, Australia, and the UK. Institutionally, Harvard University and Chinese sports and medical universities dominate, while Hong Kong institutions bridge East–West collaborations. High-impact journals like Cochrane Database emphasize clinical applications. Although Chinese scholars lead in productivity, American authors achieve higher citations.

**Conclusion:**

Over 5 decades, Wushu research has transitioned from traditional techniques to multidisciplinary health sciences, integrating geriatric care, cognitive interventions, and chronic disease rehabilitation. Strengthening international collaboration, exploring underlying mechanisms, and developing personalized digital interventions represent key future directions to enhance the global scientific impact of Chinese martial arts.

## 1. Introduction

Chinese martial arts, as an integral component of traditional Chinese culture, possess a profound historical foundation and enjoy a broad societal base.^[1,2]^ Encompassing a wide array of techniques – including barehanded forms and weapon-based practices – Chinese martial arts function not only as a cultural symbol but also embody a wealth of concepts related to physical health and well-being.^[3]^ Since the 1970s, Wushu has gradually entered the international academic discourse, emerging as a multidisciplinary research subject that integrates physical training, mental regulation, and cultural transmission^[4–9]^. In recent years, driven by national initiatives such as the “Healthy China 2030” strategy, the global promotion of cultural soft power, and increasing interdisciplinary collaboration with fields such as sports rehabilitation and public health, research on Chinese martial arts has demonstrated a marked upward trend in both volume and academic quality.

With the continuous advancement of global health and sports sciences, the physical and mental health benefits and cultural significance embedded in Chinese martial arts have garnered increasing attention from the international academic community. In particular, traditional martial art forms represented by Tai Chi have been extensively studied for their positive effects on balance enhancement, chronic disease prevention, and mental health promotion. A growing body of empirical research has highlighted the therapeutic value of Tai Chi in medical and health-related domains, demonstrating its efficacy in improving balance and reducing fall risk among older adults, alleviating chronic pain, and enhancing overall quality of life.^[10,11]^ For instance, Tai Chi interventions have been shown to significantly lower the risk of falls in elderly populations,^[12,13]^ improve pain and physical function in individuals with arthritis,^[13–15]^ and help reduce negative emotional states such as depression and anxiety.^[16]^ These findings not only underscore the health-promoting potential of Chinese martial arts but also suggest their promising integration into modern healthcare and preventive medicine. Beyond Tai Chi, other internal practices, such as Qigong, have also demonstrated beneficial effects in the prevention and management of chronic diseases and in promoting holistic health.^[17]^

Despite the growing body of empirical research, the current field remains largely fragmented. Most studies tend to focus on specific martial art styles or target populations, lacking a comprehensive and macroscopic scientometric overview. For example, Yang et al conducted a bibliometric analysis of the evidence base for Tai Chi clinical trials, but their scope was restricted solely to that particular form of martial arts.^[10]^ Similarly, Zhang et al (2020) performed a thematic bibliometric study on Qigong therapy, without incorporating other martial art modalities.^[17]^ To date, no study has systematically mapped the global research landscape of “Chinese martial arts” as a holistic category, encompassing diverse forms such as Tai Chi, Qigong, and Sanda.

To address the aforementioned research gap, this study systematically investigates the developmental trends and knowledge structure of Chinese martial arts within the international academic system from 1974 to 2025. The analysis aims to elucidate the field’s evolutionary stages, identify major research themes and their temporal trajectories, map collaboration networks among authors, institutions, and countries, and highlight influential publications and key scholars. By adopting a multidimensional perspective, this study seeks to comprehensively position Chinese martial arts within the global scientific landscape, offering theoretical support for its scientific development and international dissemination, while also providing a methodological reference for the bibliometric analysis of other traditional sport cultures. With the increasing integration of traditional martial arts and evidence-based health practices, the scientometric map constructed in this study is expected to provide valuable guidance for promoting interdisciplinary research, informing clinical applications, and facilitating the incorporation of Chinese martial arts into global strategies for preventive medicine and healthy aging.

## 2. Materials and methods

### 2.1. Data source and search strategy

This study aimed to systematically uncover the research landscape and developmental trajectory of Chinese martial arts within the international academic system. The data were sourced exclusively from the Web of Science Core Collection, including the SCI-Expanded and SSCI databases. The search covered the period from January 1974 to March 31, 2025, and was limited to articles and review papers to ensure the inclusion of studies with complete research designs and clear academic contributions.

**Figure 1.**
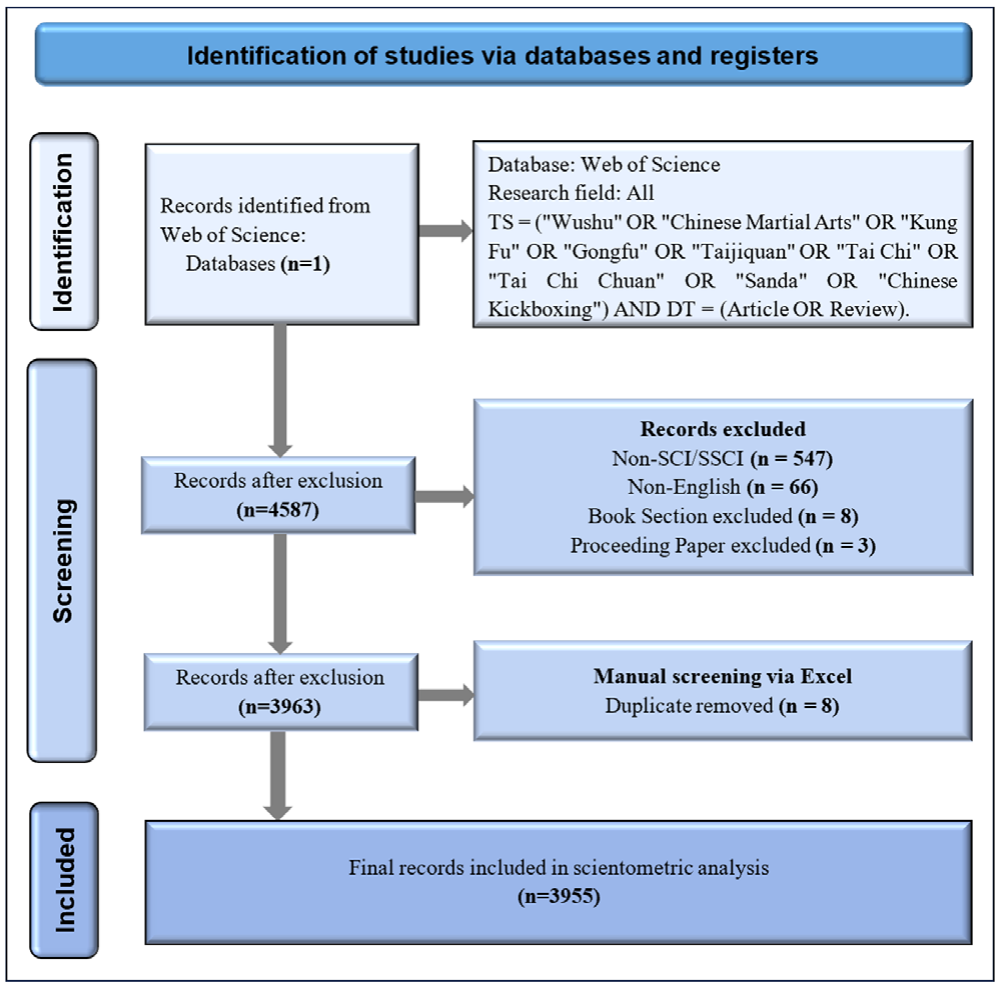
Flowchart of literature selection for scientometric analysis. A total of 4587 records were retrieved from the Web of Science Core Collection. After excluding non-SCI/SSCI, non-English, book chapters, conference papers, and duplicates, 3955 records remained for final inclusion in the analysis.

The search strategy was defined as follows: TS = (“Wushu” OR “Chinese Martial Arts” OR “Kung Fu” OR “Gongfu” OR “Taijiquan” OR “Tai Chi” OR “Tai Chi Chuan” OR “Sanda” OR “Chinese Kickboxing”) AND DT = (Article OR Review). This strategy encompassed major terminologies related to Chinese martial arts, ensuring high topical relevance and metric suitability. An initial retrieval yielded 4587 records. After excluding non-SCI/SSCI publications (n = 547), non-English articles (n = 66), book chapters (n = 8), and conference proceedings (n = 3), a total of 3963 records remained. Manual deduplication using Microsoft Excel removed 8 duplicates, resulting in a final dataset of 3955 eligible publications for scientometric analysis. The screening process is illustrated in Figure 1.

To enhance the accuracy and consistency of the retrieval process, multiple rounds of preliminary searches and keyword tests were conducted. Two researchers independently performed the final search and cross-verified the results to ensure data integrity and reliability. As this study is based exclusively on publicly available bibliometric data and does not involve human participants, ethics approval was not required.

It is noteworthy that this study did not impose language restrictions during the initial retrieval process; the search was open to all languages. However, as the Web of Science Core Collection primarily indexes international English-language journals, the final dataset predominantly comprised English publications. Chinese-language journals and theses were not included, as they are not systematically indexed by this database. Furthermore, to avoid subjective bias and data heterogeneity, this study did not incorporate other databases (e.g., PubMed, Medline), nor did it expand the dataset through manual reference list tracing. This approach was intended to ensure the objectivity of the data structure, standardization of the analytical procedures, and reproducibility of the results.

The final set of 3955 publications encompassed a wide range of research types, including interventional studies (evaluating the physical, psychological, and functional effects of martial arts), epidemiological surveys, analyses of training mechanisms and movement biomechanics, public health and rehabilitation practices, as well as empirical and review-based research on the cultural dissemination and academic development of martial arts. To ensure thematic focus, all included articles explicitly referenced Chinese martial arts in their titles, abstracts, or keywords. The full screening process is detailed in Figure 1.

To further reveal the macro-level trend of Chinese martial arts research output, basic statistical modeling was incorporated into the data analysis. Specifically, a linear regression model was applied in Microsoft Excel to examine annual publication output from 1974 to 2025, and the coefficient of determination (*R*^2^) was calculated to assess the goodness of fit of the trend line. This analysis quantitatively illustrates the trajectory of global research growth, enhancing the scientific validity and interpretability of the trend assessment.

Additionally, it should be clarified that 2 statistical counting strategies were adopted depending on the analytical dimension. The first was the actual number of publications (NP) (n = 3955) after data cleaning and deduplication, used for total output, temporal trends, and thematic analyses. The second was frequency-based counting according to author affiliations (e.g., country or institution); in coauthored papers involving multiple countries or institutions, each entity was counted once per publication. This method, resulting in higher cumulative counts than the actual number of documents, was used for analyses involving national/institutional/author productivity and collaboration networks. Distinguishing between these 2 approaches enhances metric comparability and maintains consistency across different layers of scientometric analysis.

### 2.2. Analytical tools and methods

To systematically elucidate the developmental patterns and knowledge structure of Chinese martial arts within the global scientific research system, this study employed a combination of bibliometric and visualization tools, including Bibliometrix (R 4.4.3),^[18–20]^ VOSviewer (1.6.20),^[21]^ and CiteSpace (6.2.6).^[22]^ This integrative approach facilitated the construction of a comprehensive knowledge map and identification of future research directions.

Bibliometrix is an open-source R package designed for comprehensive scientometric analysis and knowledge mapping. It has been widely adopted in bibliometric studies across various fields.^[18,19]^ In this study, Bibliometrix was primarily used for data cleaning and calculating basic bibliometric indicators, including annual publication output, annual growth rate, average annual citations, identification of prolific journals and authors, and citation metrics of influential publications. Through the Biblioshiny web interface, we generated visual representations of publication trends, journal distribution, and author productivity, including metrics such as h-index and total citations (TC). Notably, the author-level indicators used in this study – NP, TC, and h-index – were automatically computed by Bibliometrix based on Web of Science citation fields, ensuring objectivity and reproducibility.

To further explore the knowledge structure and thematic evolution of the field, VOSviewer and CiteSpace were employed to construct keyword co-occurrence networks, clustering maps, and thematic evolution timelines. Additionally, we visualized collaboration networks among countries, institutions, and authors. VOSviewer was selected for its high-quality graphical outputs, while CiteSpace’s co-occurrence algorithms, clustering techniques, and time-slicing functions enhanced the ability to identify major research topics, collaborative groups, and their dynamic trajectories.

In summary, the integrated application of Bibliometrix, VOSviewer, and CiteSpace provided comprehensive analytical support for this study, enabling a multidimensional depiction of the global research landscape on Chinese martial arts – from descriptive metrics to structural and temporal insights.

## 3. Results

### 3.1. Main information

**Figure 2.**
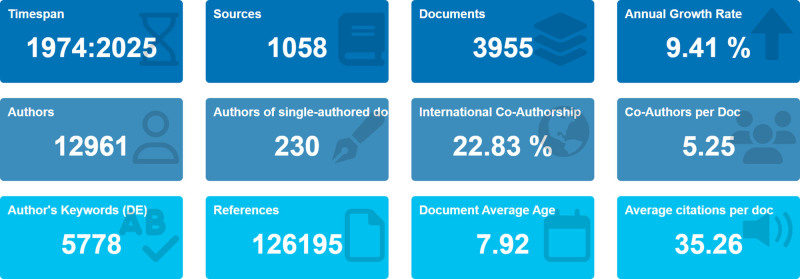
Main bibliometric indicators of Chinese martial arts research (1974–2025). Summary of key bibliometric indicators including timespan, number of documents and sources, annual growth rate, number of authors and keywords, co-authorship metrics, average citations per document, and document age, based on data from the Web of Science Core Collection.

As shown in Figure 2, a total of 3955 publications related to Chinese martial arts were indexed in the Web of Science Core Collection between 1974 and 2025, with an average annual growth rate of 9.41%. The average number of citations per document was 35.26, indicating a relatively high level of academic impact. A total of 12,961 authors were identified, with an average of 5.25 coauthors per publication; among them, 230 papers were authored by a single individual. The proportion of international co-authorship was 22.83%. The dataset included 5778 author keywords and 126,195 cited references, with the average publication age being approximately 7.92 years.

### 3.2. Annual scientific production

**Figure 3.**
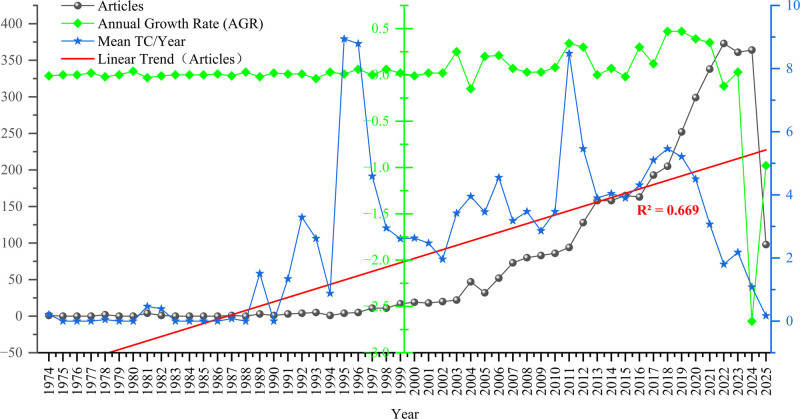
Annual publication output, growth rate, and citation frequency in Chinese martial arts research (1974–2025). Visualization of annual scientific production in the field of Chinese martial arts from 1974 to 2025. Indicators include the number of publications per year, annual growth rate, and mean total citations per year. A linear regression line (*R*^2^ = 0.669) illustrates the long-term publication trend. The chart was generated using Origin 2023 software. Data for 2025 are partial and reflect entries indexed as of March 31.

As shown in Figure 3, the scientific output in the field of Chinese martial arts from 1974 to 2025 exhibited a clear evolution from low-level dispersion to high-density concentration, with a significant upward trend in annual publications over time (*R*^2^ = 0.669). During the initial stage (1974–1995), the annual publication count remained in single digits, with only a few years before 1993 exceeding 5 publications per year, and growth rates fluctuated considerably. Between 1996 and 2005, the annual NP steadily increased from 5 to 32, and the average annual growth rate turned positive.

Since 2006, publication volume has continued to rise, surpassing 100 articles for the first time in 2012 and reaching a peak of 373 articles in 2022, with a relatively high output level around 2020. Articles published in 2011 had an average annual citation rate of 8.48. In 2023 and 2024, the NP was 361 and 364, respectively, with average annual citation rates of 2.18 and 1.08. As of March 31, 2025, 98 articles had been published, with a year-on-year growth rate of−0.98%.

### 3.3. Scientific contributions by country

In the global research landscape of Chinese martial arts, countries exhibit notable differences in terms of publication volume, academic impact, and international collaboration (see Table 1). Overall, according to publication frequency based on author affiliations, China ranks first with a total of 5259 publications, accounting for 41.9% of the global output. The United States follows with 3598 publications, representing 24.4%.

**Table 1:** Scientific production, citation impact, and international collaboration of countries in Chinese martial arts research.

Countries’ scientific production		Most cited countries			Corresponding author’s countries			
Country	Freq	Country	TC	Average article citations	Country	Articles	Articles %	MCP %
China	5259	USA	50,894	52.80	China	1656	41.9	20.6
USA	3598	China	32,835	19.80	USA	964	24.4	13.4
Australia	713	Australia	11,368	61.10	Australia	186	4.7	35.5
Canada	514	United Kingdom	6482	52.70	Canada	123	3.1	35
UK	489	Canada	4218	34.30	United Kingdom	123	3.1	33.3
Spain	286	Netherlands	2627	82.10	Korea	98	2.5	26.5
South Korea	284	Germany	2623	41.00	Spain	78	2	34.6
Germany	218	KOREA	2176	22.20	Brazil	64	1.6	31.3
Brazil	207	Spain	1975	25.30	Germany	64	1.6	29.7
Italy	176	New Zealand	1712	100.70	Italy	53	1.3	28.3
Japan	164	France	1631	46.60	Japan	50	1.3	16
France	143	Japan	1605	32.10	France	35	0.9	17.1
Netherlands	140	Italy	1496	28.20	Netherlands	32	0.8	40.6
Portugal	101	Sweden	1227	68.20	Poland	31	0.8	16.1
Poland	91	Belgium	1153	52.40	Turkey	25	0.6	8
Singapore	77	Switzerland	1145	52.00	India	23	0.6	21.7
Switzerland	74	Brazil	1083	16.90	Belgium	22	0.6	63.6
Belgium	71	Ireland	589	53.50	Switzerland	22	0.6	45.5
Sweden	67	Denmark	492	61.50	Singapore	21	0.5	28.6
Israel	65	Norway	385	32.10	Portugal	19	0.5	26.3

Article percentage refers to the proportion of publications where the corresponding author is from the listed country, relative to the total included dataset.

Freq = frequency of publications based on author affiliations, MCP = multiple country publications (percentage of articles coauthored with researchers from other countries), TC = total citations.

In terms of citations, the United States leads with 50,894 TC and an average of 52.80 citations per publication. China, while having a high publication volume, shows a total of 32,835 citations, averaging 19.80 citations per paper.

Other highly cited countries include Australia (average citations = 61.10), the United Kingdom (52.70), the Netherlands (82.10), and New Zealand (100.70). Although the Netherlands and New Zealand are not among the top 10 in publication volume, they demonstrate notably high average citation rates, indicating strong academic influence.

**Figure 4.**
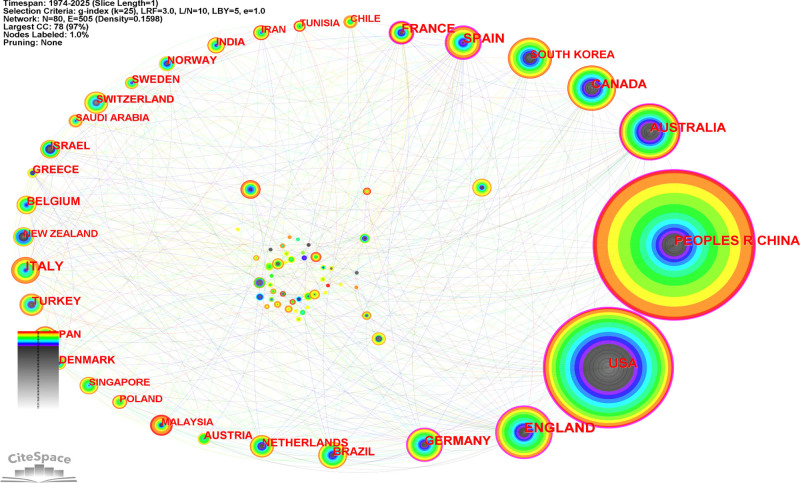
International collaboration network of countries in Chinese martial arts research (1974–2025). Each node represents a country, with node size proportional to the number of publications. Edges indicate co-authorship links between countries. Edge colors reflect the temporal sequence of collaboration (purple = earlier, red = more recent). The network is based on Web of Science Core Collection data from 1974 to 2025, with a 1-yr time slice and a g-index threshold of *k* = 25. The network consists of 80 nodes and 505 edges, with a density of 0.1598. No pruning was applied.

Figure 4 illustrates the international collaboration network among countries engaged in Chinese martial arts research from 1974 to 2025. The network comprises 80 country nodes and 505 collaborative links, with an overall density of 0.1598. China (People’s Republic of China) and the United States (USA) exhibit the largest node sizes, followed by England, Australia, Canada, and Germany. No pruning algorithm was applied, ensuring that all inter-country collaborations and structural characteristics are fully retained.

According to the Corresponding Author’s Countries data (see Table 1), China accounted for 1656 publications (41.9%), with most corresponding authors affiliated domestically. The proportion of multi-country publications (MCP%) for China was 20.6%. The United States contributed 964 papers (24.4%) with an MCP% of 13.4%.

Australia (186 publications, MCP% = 35.5%), Canada (123, MCP% = 35.0%), and the United Kingdom (123, MCP% = 33.3%) demonstrated relatively high levels of international collaboration. Countries such as Belgium (MCP% = 63.6%), Switzerland (MCP% = 45.5%), and the Netherlands (MCP% = 40.6%) had lower publication counts but higher proportions of collaborative work.

South Korea, Spain, Italy, and Japan each contributed over 50 publications, with MCP percentages ranging between 20% and 30%.

### 3.4. Institutional contributions

According to data from the Web of Science Core Collection, a total of 656 institutions were involved in Chinese martial arts research between 1974 and 2025, forming 2796 collaborative links and generating a multicentered institutional collaboration network, as shown in Figure 6. The overall network density was 0.013, indicating a relatively sparse but structurally connected collaboration landscape.

**Figure 5.**
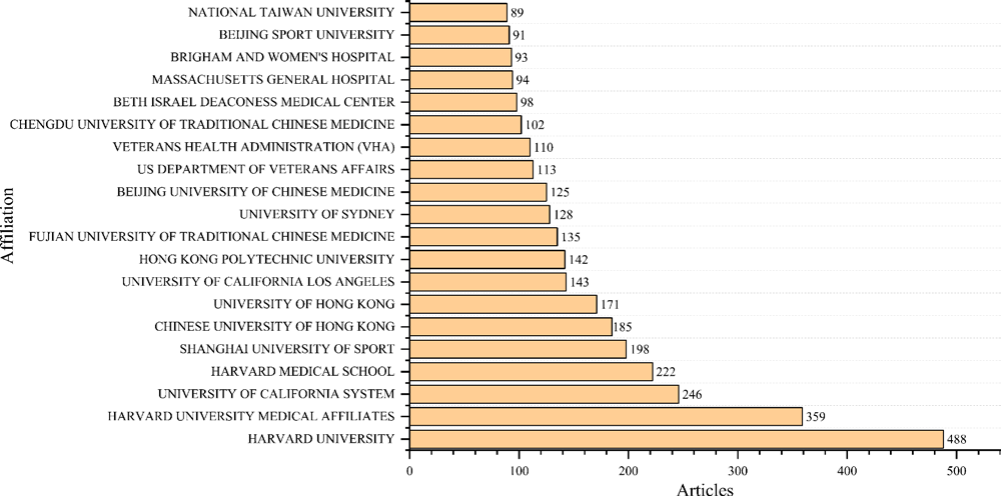
Most relevant affiliations in global Chinese martial arts research. The figure lists the top 20 institutions with the highest number of articles published in Chinese martial arts research from 1974 to 2025. The number of articles per institution is shown on the horizontal axis.

In terms of publication volume (Fig. 5), Harvard University ranked first globally with 488 articles. Several affiliated institutions also demonstrated considerable output, including Harvard Medical School (222 publications), Harvard University Medical Affiliates (359), Brigham and Women’s Hospital (93), and Massachusetts General Hospital (94). The University of California System followed with 246 publications, with the University of California, Los Angeles (UCLA) contributing significantly (143 publications).

Among institutions in mainland China, Shanghai University of Sport (198 publications), Fujian University of Traditional Chinese Medicine (135), Beijing University of Chinese Medicine (125), Chengdu University of Traditional Chinese Medicine (102), and Beijing Sport University (91) ranked among the top contributors, reflecting the active engagement of traditional sports and Chinese medicine institutions in this research domain. In Hong Kong, The Chinese University of Hong Kong (185 publications), The University of Hong Kong (171), and The Hong Kong Polytechnic University (142) each produced over 100 publications, demonstrating strong participation in international collaborations.

**Figure 6.**
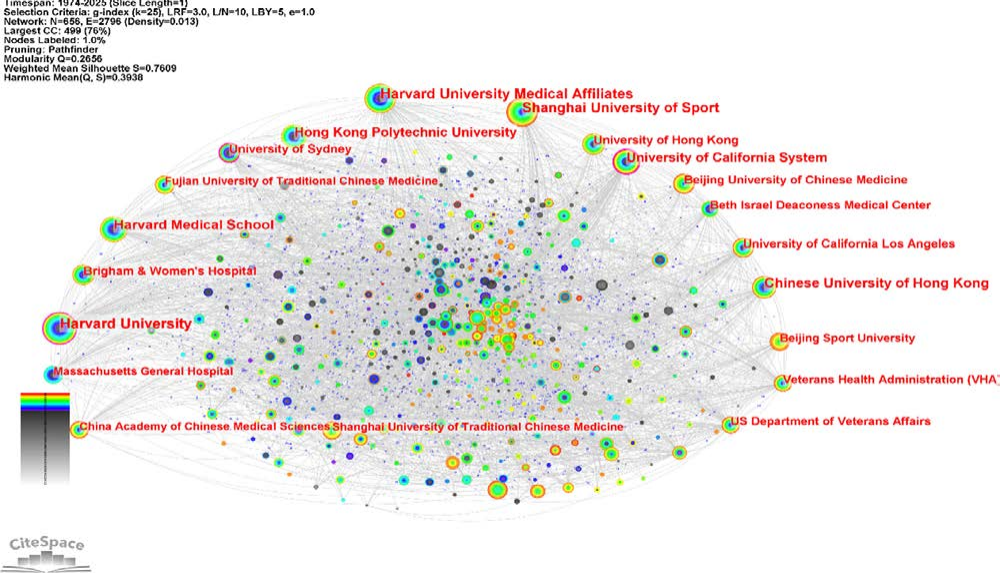
Temporal evolution of institutional collaboration in global Chinese martial arts research (1974–2025). Nodes represent research institutions, with node sizes proportional to the number of publications. Colored concentric rings indicate institutional collaboration activity over time (inner rings represent earlier years, outer rings represent recent years). Edges represent collaborations between institutions.

From the perspective of network structure (Fig. 6), institutions such as Harvard University, the University of California System, and Shanghai University of Sport exhibited larger nodes, indicating higher publication volume. The US Department of Veterans Affairs and the Veterans Health Administration (VHA) also maintained collaborative ties with multiple institutions.

The color of the links represents the chronological progression of collaborations, with red links indicating more recent partnerships, mostly emerging after 2015.

### 3.5. Journal analysis

In the global research landscape of Chinese martial arts, the publication volume and academic impact of core journals exhibit a distinct hierarchical structure (see Table 2). Evidence-Based Complementary and Alternative Medicine ranks first with 119 publications, followed by Medicine (82 publications) and the International Journal of Environmental Research and Public Health (75 publications). Although the Cochrane Database of Systematic Reviews has published only 35 articles in this field, it shows remarkable academic influence with a total of 5816 citations (TC) and an h-index of 29. Similarly, the Journal of the American Geriatrics Society and the Archives of Physical Medicine and Rehabilitation have accumulated 5756 and 3751 TC, respectively. Other journals such as PLoS ONE and Complementary Therapies in Medicine also demonstrate high productivity and visibility in this field.

**Table 2:** Publication volume, h-index, total citations, and founding year distribution of core journals in Chinese martial arts research.

Source	Articles	TC	H_index	PY_start
Evidence-Based Complementary and Alternative Medicine	119	2916	31	2004
Medicine	82	741	13	2015
International Journal of Environmental Research and Public Health	75	1394	22	2014
Complementary Therapies in Medicine	68	1430	21	2006
PLoS ONE	63	3349	30	2012
Journal of Aging and Physical Activity	55	1632	23	1998
Archives of Physical Medicine and Rehabilitation	51	3751	34	1996
Frontiers in Aging Neuroscience	51	1015	17	2011
Frontiers in Psychology	47	476	14	2012
Frontiers in Public Health	46	308	10	2017
Journal of the American Geriatrics Society	44	5756	33	1993
BMJ Open	40	402	9	2015
Complementary Therapies in Clinical Practice	38	590	15	2013
Cochrane Database of Systematic Reviews	35	5816	29	2007
BMC Geriatrics	34	628	13	2013
Trials	34	276	10	2013
Geriatric Nursing	32	479	15	2001
Archives of Budo	32	169	9	2008
American Journal of Chinese Medicine	31	1009	22	1981
Journal of Alternative and Complementary Medicine	31	1027	15	2003

This table presents the top 20 journals in global Chinese martial arts research ranked by publication volume. It summarizes their performance across 4 key metrics: number of articles, h-index, total citations, and founding year, reflecting the overall research productivity and academic impact of each journal.

PY_start = first publication year, TC = total citations.

Table 2 further illustrates the distribution of founding years of relevant journals. For instance, The American Journal of Chinese Medicine was established in 1981, while Frontiers in Public Health began in 2017, indicating both continuity and expansion in the range of journals publishing Chinese martial arts research over time.

### 3.6. Author-level contributions

#### 3.6.1. Identification of the most productive authors

**Figure 7.**
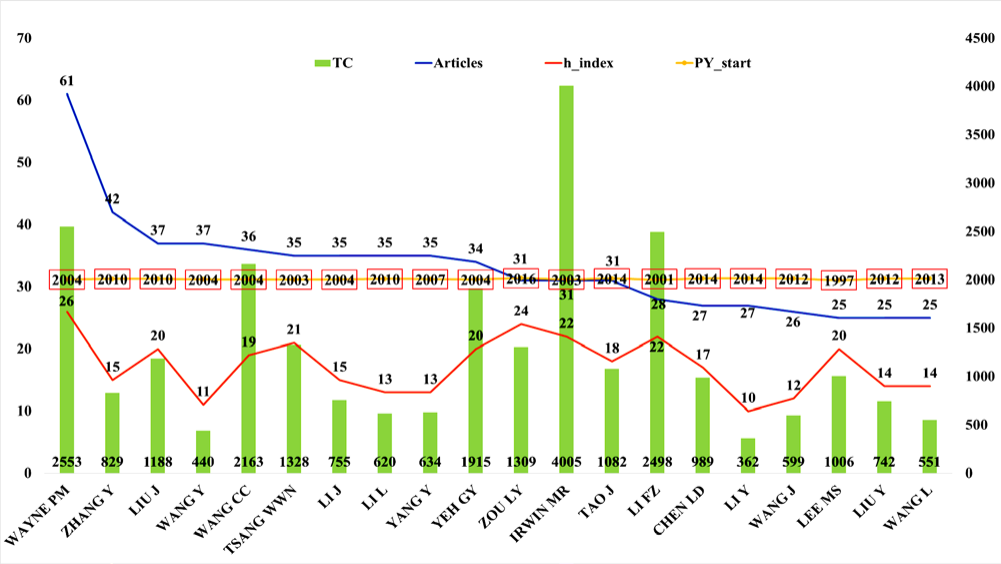
Scientometric profile of the top productive authors in Chinese martial arts research, including publication count, total citations, h-index, and first year of publication. The figure presents the top 20 authors ranked by the number of publications. 4 key indicators are displayed: number of publications (Articles), total citations (TC), h-index, and the year of first publication (PY_start). The data were extracted from the Web of Science Core Collection and visualized using Microsoft Excel.

Based on publication volume, a total of 20 prolific authors in the field of Chinese martial arts research were identified (Fig. 7). Among them, Wayne PM ranked first with 61 publications, followed by Zhang Y (42 articles), Wang Y and Liu J (37 articles each), and Wang CC (36 articles). Most of these authors began publishing in this field in the mid-2000s. Some, such as Wang Y and Li J, have maintained a consistent output since 2004, while others, including Liu J and Li L, became active after 2010.

#### 3.6.2. Author impact evaluation

To further identify the most influential authors in the field of Chinese martial arts research, this study analyzed highly productive authors based on 2 key indicators: TC and h-index. The results revealed that Irwin MR (TC = 4005, h-index = 22) and Wolf SL (TC = 3304, h-index = 20) stood out in terms of citation volume, indicating high visibility and academic impact within the field. Although Wayne PM had the highest NP, his TC (2553) and h-index (26) also reflected a strong alignment between productivity and impact. Other authors, such as Li FZ (TC = 2498, h-index = 22) and Harmer P (TC = 2307, h-index = 19), also demonstrated consistent academic influence.

**Figure 8.**
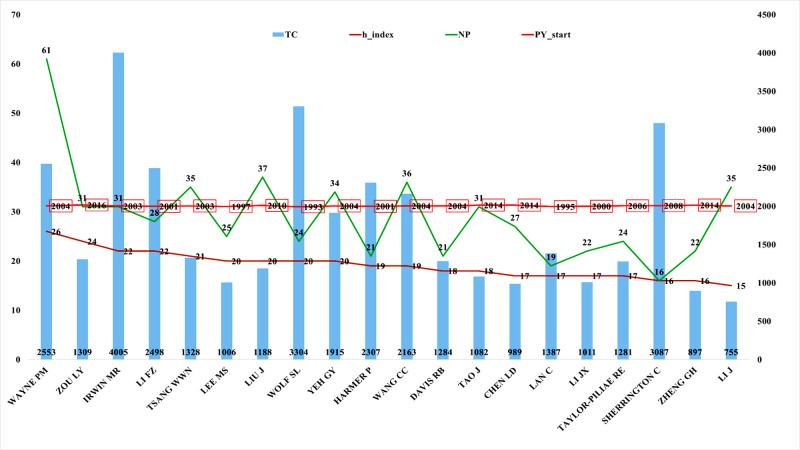
Integrated bibliometric indicators of influential authors in Chinese martial arts research (h-index, number of publications, total citations, and initial year of publication). The figure highlights the top 20 influential authors based on total citations (TC) and h-index, complemented by number of publications (NP) and first publication year (PY_start). The data were extracted from the Web of Science Core Collection and visualized using Microsoft Excel.

Figure 8 visualizes the relationship among TC, publication count, and h-index for the selected authors, helping to identify scholars with both high productivity and high impact. As shown, Irwin MR achieved the highest TC with a moderate NP, while Lee MS and Yeh GY, though not among the top 5 in publication count, exhibited high h-index and citation scores, suggesting strong impact per output.

#### 3.6.3. Author collaboration network structure

**Figure 9.**
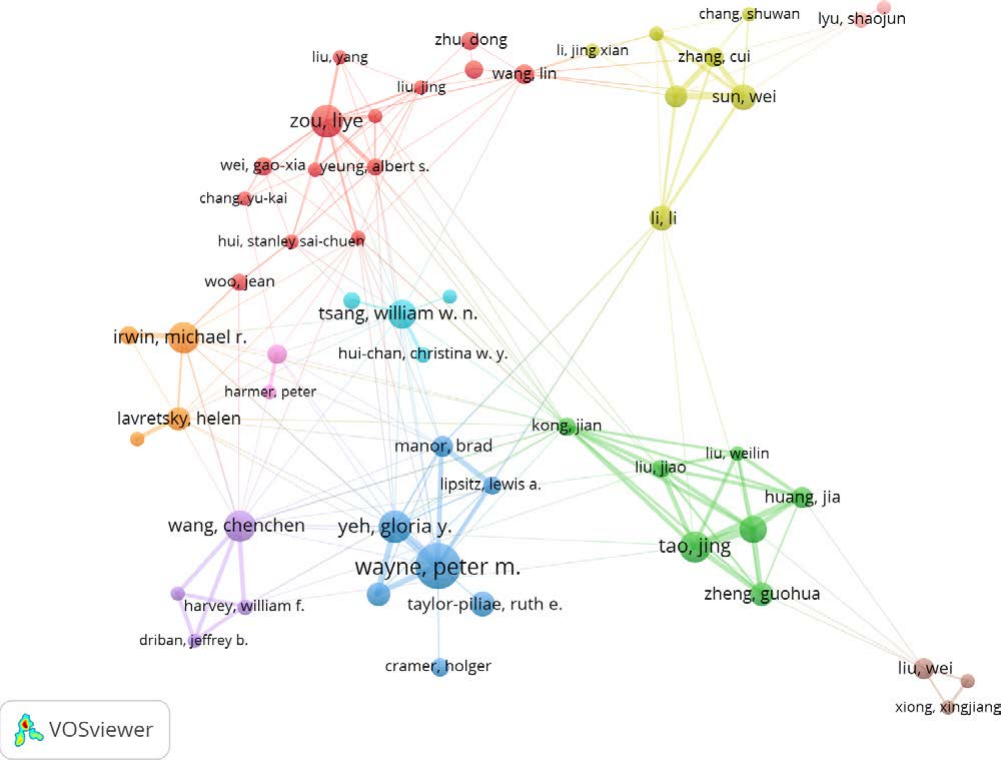
Author collaboration network in Chinese martial arts research (1974–2025). Nodes represent individual authors, with node size proportional to the number of publications. Edges indicate the frequency of co-authorship, and colors distinguish different collaboration clusters. The network was generated using VOSviewer based on data from the Web of Science Core Collection (1974–2025). A minimum publication threshold of 10 was applied, resulting in the inclusion of 69 authors.

Based on the author collaboration network generated using VOSviewer (Fig. 9), a total of 69 authors with at least 10 publications were included to map the core scholarly collaboration patterns in Chinese martial arts research. In the network, nodes represent individual authors, with node size proportional to publication volume, edges indicating co-authorship links, and colors distinguishing different collaboration clusters.

Several relatively stable research clusters can be identified. The blue cluster led by Wayne PM is closely connected to Yeh GY and Taylor-Piliae RE; the green cluster centered around Tao, Jing includes collaborators such as Zheng, Guohua, forming a sizable research group. Zou L, together with Yeung AS, forms a distinct red cluster indicating a stable partnership. Similarly, Li L and Sun W, as well as Irwin MR and Lavretsky H, exhibit independent collaborative subgroups.

In addition, some authors (e.g., Tsang, William WN) serve as bridges between multiple clusters, facilitating cross-group collaborations and indicating a growing trend toward interdisciplinary and inter-institutional research integration.

### 3.7. Keyword co-occurrence and clusters

#### 3.7.1. Author keyword co-occurrence analysis

Keyword co-occurrence analysis is instrumental in identifying core terminologies and thematic focuses within a research field. Based on data extracted from the Web of Science (1974–2025), Figure 10 presents the co-occurrence network of author keywords in Chinese martial arts research. The network comprises 515 nodes and 6217 links, with a density of 0.047, indicating a relatively high degree of keyword connectivity and thematic concentration.

**Figure 10.**
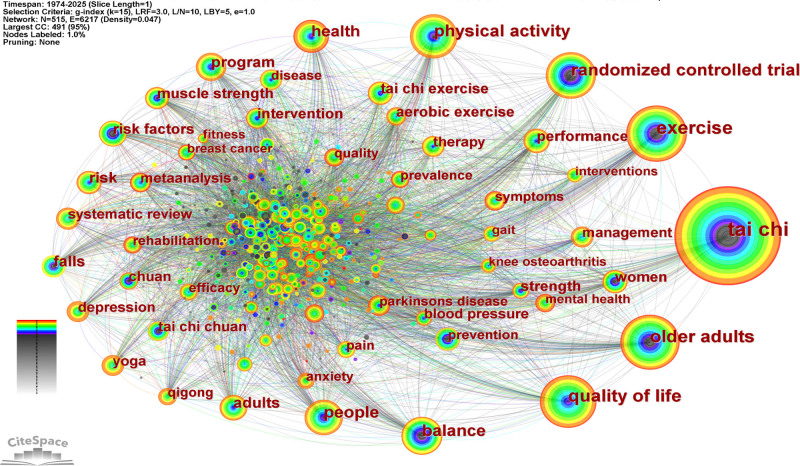
Keyword co-occurrence network in Chinese martial arts research (1974–2025). Keyword co-occurrence network generated using CiteSpace (version 6.2.R6) based on data from the Web of Science Core Collection (1974–2025). Each node represents a keyword, with node size indicating frequency of occurrence. Edges denote co-occurrence relationships between keywords. Color layers reflect the temporal distribution of keywords (purple = earlier; red = recent). The network includes 515 nodes and 6217 edges, with a g-index threshold of *k* = 15 and no pruning applied.

High-frequency keywords include “tai chi” (2107 occurrences), “exercise” (918), “older adults” (803), “quality of life” (791), and “physical activity” (610). The term “tai chi” also demonstrated the highest betweenness centrality (0.07), positioning it at the core of the network. Other frequently occurring terms include “randomized controlled trial” (533), “balance” (507), and “falls” (256).

In terms of temporal distribution, keywords such as “quality of life,” “older adults,” and “physical activity” began to appear more frequently in the late 1990s. In recent years, mental health-related terms such as “depression,” “anxiety,” and “self-esteem” have also emerged within the network, reflecting an expansion and evolution of the thematic structure.

#### 3.7.2. Cluster and thematic evolution mapping (CiteSpace visualization)

**Figure 11.**
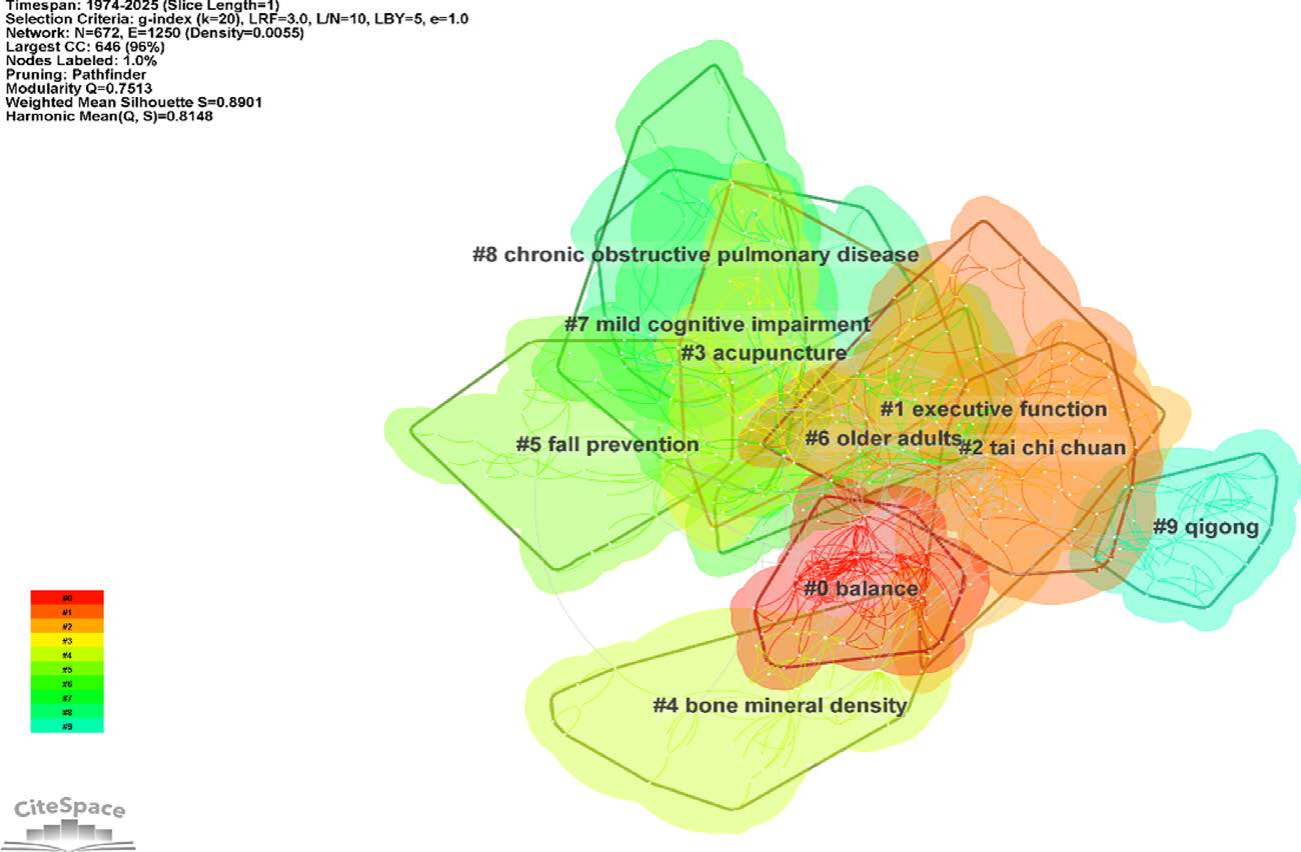
Keyword co-occurrence clusters in global Chinese martial arts research (1974–2025). This figure presents the keyword co-occurrence clustering network of Chinese martial arts research based on Web of Science Core Collection data, visualized using CiteSpace. Nodes represent keywords, with node size indicating co-occurrence frequency and color reflecting the first year of appearance (from red to green). The 10 largest clusters (#0–#9) are labeled with their dominant themes, such as “balance,” “executive function,” and “tai chi chuan.” The network achieved a modularity Q of 0.7513 and a silhouette score of 0.8901, reflecting strong structural clarity and cluster cohesion.

To further explore the research hotspots and underlying knowledge structure of Chinese martial arts studies worldwide, this study employed CiteSpace to conduct a keyword co-occurrence clustering analysis of publications indexed in the Web of Science Core Collection from 1974 to 2025. As shown in Figure 11, the resulting co-occurrence network comprises 672 nodes and 1250 links, with a modularity *Q* value of 0.7513 and a weighted mean silhouette score of 0.8901, indicating a high level of structural differentiation and internal homogeneity among clusters.

Ten major clusters were extracted and analyzed, representing the most prominent thematic groups within the field. These include: #0 “balance,” #1 “executive function,” #2 “tai chi chuan,” #3 “acupuncture,” #4 “bone mineral density,” #5 “fall prevention,” #6 “older adults,” #7 “mild cognitive impairment,” #8 “chronic obstructive pulmonary disease,” and #9 “qigong.” Cluster #0 demonstrated the highest network centrality. Clusters #1 and #7 relate to cognitive performance, while clusters #2 and #9 are associated with traditional martial arts practices such as Tai Chi and Qigong. Clusters #4 and #5 focus on health-related outcomes like bone density and fall prevention, and cluster #8 is centered on chronic respiratory conditions.

#### 3.7.3. Research frontiers and future trend prediction

**Figure 12.**
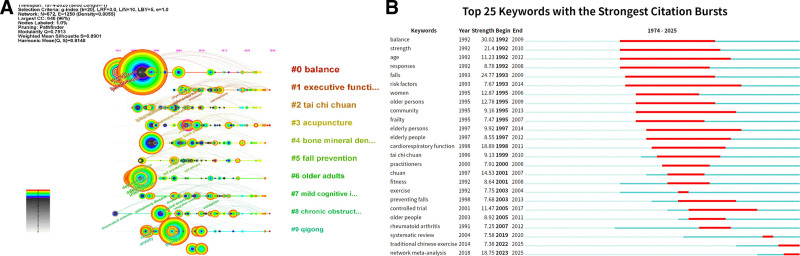
Temporal evolution and burst strength of keywords in Chinese martial arts research (1974–2025). (A) Timeline view of keyword clusters. (B) Top 25 keywords with strongest bursts and their time spans.

To illustrate the temporal evolution of research hotspots in Chinese martial arts, this study constructed both a thematic evolution pathway (Fig. 12A) and a keyword burst detection map (Fig. 12B), based on keyword clustering and burst term analysis. These visualizations reflect shifts in research priorities and terminology from 1974 to 2025.

Figure 12A presents the temporal evolution of the 10 major keyword clusters, namely: #0 “balance,” #1 “executive function,” #2 “tai chi chuan,” #3 “acupuncture,” #4 “bone mineral density,” #5 “fall prevention,” #6 “older adults,” #7 “mild cognitive impairment,” #8 “chronic obstructive pulmonary disease,” and #9 “qigong.” In this map, each node represents a keyword, with node size indicating co-occurrence frequency and color representing the time of first appearance. Notably, clusters #0, #2, and #4 are primarily distributed between the 1990s and 2000s, while clusters #1, #7, and #9 have emerged more prominently since 2020, suggesting recent thematic transitions in the field.

Figure 12B displays the top 25 keywords with the strongest citation bursts and their respective time spans. The term “balance” exhibited the highest burst strength (30.02, 1992–2009), followed by “falls” (24.77, 1993–2009), “strength” (21.40, 1992–2010), and “cardiorespiratory function” (18.89, 1998–2011). More recent emergent terms include “systematic review” (2020), “traditional Chinese exercise” (2022–2025), and “network meta-analysis” (2023–2025).

Taken together, Figure 12A and B reveals a consistent temporal pattern in keyword evolution, highlighting distinct periods of thematic concentration and shifts in scholarly focus over the past 5 decades.

### 3.8. Three-field plot analysis of authors, institutions, and countries

**Figure 13.**
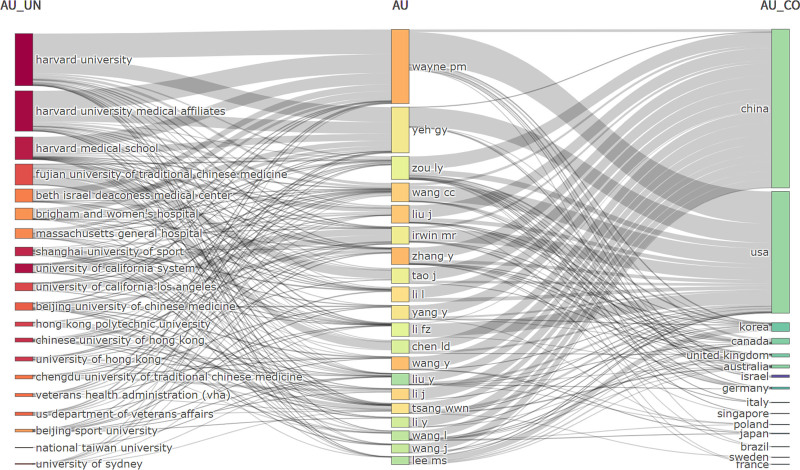
Three-field collaboration network (author–institution–country) in global Chinese martial arts research (1974–2025). This figure presents the three-field plot illustrating the relationships among authors (AU), institutions (AU_UN), and countries/regions (AU_CO) based on publications indexed in the Web of Science Core Collection. The left field represents author affiliations (institutions), the center shows high-yield authors, and the right field indicates the corresponding countries or regions. Links between elements reflect collaboration or affiliation, with link thickness representing the frequency of co-authorship or institutional–national connections.

Figure 13 illustrates the three-field plot of author (AU), institution (AU_UN), and country (AU_CO) collaborations in global Chinese martial arts research. In this diagram, authors are positioned in the central column, with affiliated institutions on the left and countries on the right.

At the author level, Wayne PM, Yeh GY, Zou LY, Wang CC, and Liu J are identified as the most prolific contributors in the field. Their research has predominantly focused on the application of Tai Chi in chronic disease management, mental health, and rehabilitation. Notably, Wayne PM maintains close collaborations with several leading U.S. medical research institutions.

At the institutional level, Beijing Sport University, Chengdu University of Traditional Chinese Medicine, Fujian University of Traditional Chinese Medicine, and The Hong Kong Polytechnic University are among the top publishing institutions in Greater China. In the United States, Harvard University, Massachusetts General Hospital, and Brigham and Women’s Hospital stand out as frequent contributors. Strong collaborative ties are observed among institutions in mainland China, Hong Kong, and the United States. Key institutions in Hong Kong include The Chinese University of Hong Kong and The University of Hong Kong.

At the country level, China and the United States are the leading contributors in terms of publication volume and international collaboration. Other countries actively involved in global partnerships include the United Kingdom, Australia, Canada, South Korea, Israel, Sweden, and Singapore.

## 4. Discussion

This study conducted a comprehensive review and analysis of global research on Chinese martial arts from 1974 to 2025, using bibliometric methods based on the Web of Science database. The results reveal that scientific inquiry into Chinese martial arts has undergone a clear evolution from a slow emergence to a period of rapid growth, with a notable surge in scholarly output over the past decade. This upward trend aligns with the global rise of traditional exercise therapies,^[23]^ highlighting the renewed vitality of cultural heritage practices within the context of modern scientific discourse. In particular, Tai Chi, as the most representative form of Chinese martial arts, has become the dominant research focus and has established a certain level of influence in international medical and exercise science communities.^[10]^ This phenomenon can be explained from both supply and demand perspectives. On the one hand, the global aging population and the prevalence of chronic diseases have created substantial demand for safe, low-cost, and highly feasible physical activity interventions – characteristics that traditional martial arts practices like Tai Chi inherently possess.^[24]^ On the other hand, a growing body of evidence-based studies has consistently confirmed the efficacy of Tai Chi interventions for improving health outcomes, such as fall prevention,^[11,12]^ joint function,^[25,26]^ cardiorespiratory endurance,^[27]^ and mental health conditions including depression and anxiety.^[28,29]^ This accumulating body of scientific evidence has increased the credibility and acceptance of martial arts therapies among both academic and clinical communities. As a result, Tai Chi has been recognized in official reports by institutions such as the U.S. National Institutes of Health and European sports medicine guidelines for its benefits in elderly health promotion.^[10]^ Moreover, several national guidelines for chronic disease management have begun to include Tai Chi and Qigong as recommended exercise therapies.^[30]^ In this sense, the global development of Chinese martial arts research serves as a paradigmatic example of how traditional health practices can be successfully integrated with modern scientific methodologies to address pressing human health needs.

### 4.1. Evolutionary trends and developmental phases

Based on the bibliometric findings, the development of scientific research on Chinese martial arts can be broadly divided into 3 distinct phases, each characterized by specific features and driving forces.

Initiation Phase (1970s–1990s): During this period, martial arts-related publications were sparse and thematically scattered, mostly consisting of case reports or qualitative observations, with little systematic investigation. This can be attributed to 2 main reasons: first, martial arts were traditionally transmitted as practical skills, and thus lacked integration with scientific research paradigms; second, martial arts were largely viewed as cultural or competitive activities, and their relevance to medical research was not widely recognized. In Western scientific discourse at the time, practices like Tai Chi were often considered “Eastern mystical wellness arts” rather than credible health interventions. A representative early contribution is the experimental study by Jin, which explored the physiological and emotional effects of Tai Chi practice. The study found that Tai Chi could increase heart rate, enhance norepinephrine excretion, lower cortisol levels, and improve emotional states.^[31]^ These early efforts laid foundational groundwork for future exploration.

Expansion Phase (2000s): In the early 21st century, driven by the emergence of concepts such as “integrative medicine” and “exercise prescription,” martial arts began to be more frequently incorporated as intervention modalities in clinical research. This phase was supported by Chinese government initiatives (e.g., the inclusion of Tai Chi in national fitness programs) and a growing Western interest in complementary and alternative medicine.^[32]^ Numerous randomized controlled trials (RCTs) were launched during this period, covering a range of conditions such as arthritis, cardiac rehabilitation, diabetes, and fall prevention among the elderly.^[32–34]^ These high-quality studies substantially improved the academic credibility of martial arts-based research.

Specifically, Wolf et al^[35]^ reported that a 15-week Tai Chi intervention led to a 47.5% reduction in the risk of multiple falls, while also improving physiological and psychological indicators such as blood pressure regulation and fear of falling. Song et al,^[36]^ in a randomized controlled trial involving elderly women with osteoarthritis, found that 12 weeks of Sun-style Tai Chi significantly alleviated joint pain and stiffness, improved balance and abdominal muscle strength, and reduced perceived difficulties in physical functioning, whereas the control group showed no improvements or even experienced functional decline during the same period. Li et al^[37]^ conducted a 6-month RCT including 256 older adults and found that the Tai Chi group significantly outperformed the control group in reducing fall frequency and fall-related injuries, as well as in improving functional balance (e.g., Berg Balance Scale and one-leg standing tests), with a 55% reduction in fall risk and sustained effects after 6 months. Later, Li et al^[38]^ conducted another RCT among patients with Parkinson disease, confirming that 24 weeks of Tai Chi training significantly enhanced balance control (as measured by maximum stability and directional control), and outperformed resistance training and stretching groups in gait stride, functional reach, and fall incidence, with the intervention effects remaining significant 3 months post-intervention. These studies not only provide strong clinical evidence but also expand the application scope of Chinese martial arts in neurodegenerative diseases and elderly health management.

Deepening Phase (2010s–present): The past decade has marked a period of deepening and diversification. On one hand, a large number of meta-analyses and systematic reviews have summarized the effects of Tai Chi, Qigong, and related practices across various populations and health outcomes,^[39–42]^ confirming their benefits while also noting limitations (e.g., small sample sizes or methodological heterogeneity). On the other hand, research themes have expanded, delving into the underlying mechanisms, long-term adherence, and psychosocial benefits of martial arts interventions.^[24,43]^ A particularly notable trend is the surge in interdisciplinary research. For example, some studies now incorporate neuroimaging techniques to examine structural and functional changes in the brain following Tai Chi practice^[44]^; others employ biomarkers to assess the regulatory effects of Qigong on immune and inflammatory responses.^[45]^ More recently, martial arts have been integrated with emerging technologies such as virtual reality and artificial intelligence (AI) to develop innovative intervention models.^[46]^ These developments reflect a paradigm shift in the field – from merely answering “does it work” to investigating “why and how does it work.”

In sum, the progression of martial arts research illustrates a trajectory of increasing scientific integration – from marginality to mainstream, from experiential practice to evidence-based validation, and from macro-level phenomena to micro-level mechanisms. This evolution not only signifies methodological advancement but also reflects sociocultural drivers, as traditional Chinese martial arts become more globally accepted through the lens of scientific legitimacy.^[47]^ With the participation of new-generation researchers and the advancement of research methodologies, Chinese martial arts scholarship is poised to enter a more mature and stable phase, contributing to the development of a theoretically grounded, evidence-informed exercise intervention framework with Chinese characteristics.

### 4.2. Research hotspots and multidisciplinary integration

The bibliometric analysis reveals that current research on Chinese martial arts is highly concentrated in the domains of health promotion and disease rehabilitation. This focus aligns closely with the core traditional value of martial arts – strengthening the body and preserving health – and is consistent with the global priorities in health sciences. According to high-frequency keywords and clustering results, major research themes include: balance/fall prevention, arthritis/bone mineral density, cognitive function/Alzheimer disease, chronic disease rehabilitation (e.g., COPD, heart failure), and mental health disorders such as depression and anxiety. These topics are closely tied to typical geriatric and chronic disease-related health issues, indicating a high degree of alignment between martial arts research and the health demands of an aging global population.^[30]^ Take fall prevention as an example: the World Health Organization (WHO) has classified falls among older adults as a major public health concern, and physical activity interventions are considered one of the most effective prevention strategies.^[48]^ Tai Chi, through enhancing lower limb strength, improving vestibular function, and stimulating proprioception, has been shown to significantly reduce fall risk among the elderly and to improve balance control in individuals with Parkinson disease.^[38,49]^ Based on robust clinical evidence, Tai Chi training programs have been widely integrated into community-based balance interventions for older adults in many countries.^[50]^ This not only underscores the practical value of martial arts in public health but also reflects their transition from cultural heritage to evidence-based intervention tools within modern healthcare systems.

In the realm of mental health, many individuals with chronic diseases also experience comorbid psychological conditions, such as depression, anxiety, and insomnia. Identifying non-pharmacological approaches to improve patients’ psychological well-being has become a major focus in medical research. Owing to their unique combination of physical activity and meditative relaxation, Tai Chi and Qigong are increasingly recognized as mind-body exercises that promote psychophysiological integration. Numerous studies have reported their positive effects in alleviating symptoms of depression, anxiety, and sleep disturbances.^[51]^ Meta-analyses indicate that Tai Chi can significantly reduce depression scores and anxiety levels in middle-aged and older adults (standardized mean difference ≈ –0.5 to–0.7, *P* < .001), with effects comparable to those of conventional aerobic exercise.^[52]^ As a result, Tai Chi is now being increasingly incorporated into comprehensive mental health intervention programs, particularly as a psychological support modality for elderly patients with chronic conditions.^[29]^

Cognitive function has also emerged as a prominent research hotspot in recent years. Mild cognitive impairment, regarded as a high-risk stage for Alzheimer disease, is a key target for preventive interventions. Exercise is known to slow the progression of cognitive decline, and Tai Chi – requiring memory of movement sequences, sustained attention, and motor coordination – has been hypothesized as an ideal intervention for cognitive enhancement. Recent systematic reviews have shown that Tai Chi practiced for 12 weeks or longer can significantly improve global cognitive performance and executive function in older adults with mild cognitive impairment,^[53]^ providing evidence-based support for its application in this population. Furthermore, RCTs have begun exploring the impact of Tai Chi on early-stage Alzheimer patients, including the use of neuroimaging techniques to observe changes in brain structure and function.^[54]^ These interdisciplinary studies are expected to enrich our understanding of the underlying mechanisms by which Tai Chi may influence cognitive outcomes.

In addition to the diversification of research themes, the academic dissemination platforms of Chinese martial arts have exhibited a distinct stratified structure and evolving trajectory, further reflecting the expanding scope of interdisciplinary integration in this field. The current journal ecosystem reveals a dual-layer pattern characterized by both “high productivity” and “high impact.” For example, Evidence-Based Complementary and Alternative Medicine, Medicine, and the International Journal of Environmental Research and Public Health lead in publication volume, spanning disciplines such as traditional medicine, public health, and exercise science. In contrast, although journals such as the Cochrane Database of Systematic Reviews and the Journal of the American Geriatrics Society publish fewer articles, they demonstrate substantial academic influence in domains like systematic reviews, rehabilitation medicine, and geriatrics due to their high citation rates and h-index values. Overall, the dissemination landscape of martial arts research is evolving into a decentralized structure rooted in complementary and alternative medicine, while progressively extending toward rehabilitation and health promotion.

From a temporal perspective, the evolution of journals also reveals a shift from traditional to modern scholarly platforms. Early journals, such as The American Journal of Chinese Medicine (established in 1981), laid the foundation for international communication of martial arts research. In recent years, the rapid emergence of open-access journals such as Frontiers in Public Health and Complementary Therapies in Medicine has expanded dissemination channels, enhancing global visibility and interdisciplinary reach. These trends indicate a gradual integration and reconstruction of Chinese martial arts within contemporary scientific paradigms.

In summary, disciplines such as sports science, rehabilitation medicine, geriatrics, psychology, and neuroscience have increasingly converged in the study of martial arts. This interdisciplinary collaboration has facilitated a paradigm shift from traditional cultural practices to evidence-based health interventions. Looking ahead, the involvement of emerging fields such as bioinformatics and sports biomechanics may enable breakthroughs at the mechanical, biomarker, and molecular levels. For instance, the use of wearable sensors and artificial intelligence can enable fine-grained assessments of Tai Chi movements, thereby elucidating their specific effects on balance control. Similarly, the application of epigenetic tools may uncover how practices like Qigong modulate gene expression associated with aging. These advancements will provide a robust foundation for translating martial arts into digital health strategies and precision medicine, thereby expanding their practical value in future healthcare applications.

### 4.3. Strengths, limitations, and future directions

Based on the findings of this scientometric analysis, the global research on Chinese martial arts demonstrates several notable strengths while also presenting areas for improvement to inform future directions. First, the growing body of evidence-based research constitutes a major advantage. An increasing number of high-quality RCTs and systematic reviews support the efficacy of martial arts interventions.^[10]^ In recent years, international research on Tai Chi and related traditional martial arts has intensified, with evidence-based support significantly strengthened. Existing systematic reviews have shown that Tai Chi plays a positive role in improving cognitive function, cardiorespiratory endurance, mental health, and chronic disease management.^[14,55,56]^ In the past few years, a growing number of RCTs and meta-analyses have further expanded its application scope. Li et al^[57]^, through a Bayesian network meta-analysis, identified significant cognitive benefits of Baduanjin and Tai Chi. Siu et al in 2021 reported in a large-scale RCT that Tai Chi led to sustained improvements in sleep quality among older adults.^[58]^ Allen et al in 2025 emphasized in their review that Tai Chi stands out as one of the most evidence-supported non-pharmacological therapies for osteoarthritis and is now widely recognized as a leading mind-body intervention.^[59]^ Other studies have further confirmed its potential in alleviating cancer-related fatigue^[60]^; improving VO_2_ max^[61]^; regulating autonomic nervous function^[62]^; and enhancing body composition among adolescents.^[63]^ These findings suggest a wide range of applications across diverse populations and functional domains. As a result, Chinese martial arts have become one of the few traditional exercise therapies included in Western clinical guidelines. Tai Chi, in particular, has been recommended by the American Geriatrics Society for fall prevention, as noted in the guidelines published between 2001 and 2010,^[64–66]^ and has also been incorporated into the 2012 and 2019 osteoarthritis management guidelines issued by the American College of Rheumatology, where it is endorsed as a core non-pharmacological treatment for patients with knee and hip osteoarthritis.^[67,68]^ Second, current research themes align closely with pressing public health needs. International research hotspots focus predominantly on healthy aging and chronic disease management. Tai Chi, for instance, offers a safe and effective exercise modality for older adults, with proven benefits in reducing healthcare burden and enhancing quality of life.^[69]^ Third, martial arts research exhibits a high degree of international engagement. While China and the United States lead in research output, over 80 countries have contributed to this field, reflecting growing global recognition and validation of the health value of Chinese martial arts.^[9]^ Notably, countries such as Australia and Canada have not only fostered local academic interest in Tai Chi but have also integrated martial arts into community-based rehabilitation programs.^[14]^ This broad and diverse international participation has infused the field with cross-cultural perspectives and theoretical innovation. Overall, nearly 5 decades of research have led to the establishment of a research paradigm that integrates both Western scientific methodologies and traditional Chinese practices. This evolving framework emphasizes rigorous experimental design while respecting the cultural specificity of traditional martial arts, laying a solid foundation for advancing the depth and scope of future investigations.

Despite the notable progress achieved, several critical challenges persist in current research on Chinese martial arts. Chief among them is the issue of methodological heterogeneity. Although a substantial number of RCTs have been conducted, considerable variation remains in intervention modalities, movement styles, training frequency, and duration, thereby compromising the comparability and reproducibility of study outcomes.^[70]^ For instance, the existence of multiple Tai Chi schools leads to significant variation in the forms used across studies.^[71]^ A systematic review on knee osteoarthritis highlighted that different Tai Chi styles yielded inconsistent effects on functional improvement.^[72]^ These discrepancies underscore the need to standardize intervention protocols – such as defining core movement components, session duration, and frequency – to enhance the scientific rigor and generalizability of evidence-based studies. Second, there is a relative lack of mechanistic research. Existing literature predominantly focuses on “whether” martial arts interventions are effective, while insufficient attention is paid to “how” they exert their effects. This gap limits the theoretical credibility of martial arts therapy within modern biomedical paradigms. For example, while Tai Chi has been shown to alleviate depression, its underlying neurophysiological mechanisms remain unclear. Some scholars hypothesize involvement of the hypothalamic-pituitary-adrenal (HPA) axis and autonomic nervous system regulation, but direct experimental validation is lacking.^[73]^ Similarly, practices such as Qigong are often described in traditional terms such as “regulating qi and nourishing the mind,” yet their physiological foundations – possibly involving vagal nerve activation or changes in brainwave activity – are not well substantiated by empirical data. To address these gaps, future research should incorporate advanced techniques such as functional magnetic resonance imaging (fMRI), neuroelectrophysiology, and biomarker monitoring to elucidate the biological and psychological mechanisms underlying martial arts interventions. Such efforts will strengthen the scientific legitimacy of Chinese martial arts and enhance their global acceptance as evidence-based health practices.

Furthermore, the current scope of research on Chinese martial arts remains limited. International academic inquiry in this field is predominantly focused on Tai Chi and Health Qigong, while scientific investigations into other forms – such as Sanda (Chinese kickboxing) or traditional weapons-based routines – are sparse. This imbalance may partly stem from the inherent difficulty of designing RCTs for combat-oriented disciplines, yet it also reflects a broader neglect of martial arts diversity. In fact, practices such as Sanda may offer unique benefits for youth development, particularly in enhancing psychological resilience and self-defense capabilities, thus warranting exploration from the perspectives of sports sociology or educational science. Likewise, the performative and aesthetic aspects of martial arts routines may be examined in relation to psychological constructs such as self-efficacy and social participation.^[74]^ Therefore, future research should be encouraged to incorporate both competitive and performance-oriented elements of martial arts – within safe and ethically sound frameworks – to enrich the dimensionality of martial arts scholarship.

Finally, it is important to acknowledge the regional and publication biases that may affect this field. Since the present study relies solely on the Web of Science Core Collection, a substantial body of research indexed in other international databases and Chinese-language sources was not included. This may introduce a degree of publication bias, particularly in underestimating the domestic prominence of certain research topics within China. For instance, traditional Chinese exercise forms such as Baduanjin and Wuqinxi have been widely studied in China but are underrepresented in English-language publications. Similarly, topics related to the cultural dissemination or historical evolution of martial arts are often published in Chinese and thus fall outside the scope of this scientometric analysis. To address this limitation, we recommend future studies to integrate data from Chinese-language databases such as CNKI (China National Knowledge Infrastructure) to provide a more comprehensive and balanced global overview – encompassing both Western and Eastern academic perspectives. Nevertheless, we contend that English-language literature sufficiently reflects international research trends, particularly in the domains of natural science and medical research.

Based on the above analysis, future research on Chinese martial arts can be deepened and expanded along several key directions: First, there is a pressing need to strengthen mechanistic investigations by employing multidisciplinary approaches to elucidate the underlying pathways of martial arts interventions – such as neural regulation, endocrine modulation, and immune responses. For example, neuroimaging techniques could be used to reveal how Tai Chi influences brain functional connectivity, while changes in inflammatory markers or stress-related hormones before and after practice could provide insight into physiological mechanisms.^[75]^ Second, enhancing research design quality is critical. Future studies should promote high-quality RCTs through multicenter and international collaborations. Integrating these with systematic reviews and meta-analyses will improve the strength and grade of evidence. Notably, large-scale RCTs on Tai Chi for fall prevention, jointly conducted by institutions in China and the United States, could serve as a benchmark. Third, innovation in intervention delivery is essential. This includes integrating martial arts with modern technologies – for instance, developing motion monitoring systems based on wearable sensors or embedding Tai Chi practice into telehealth and virtual reality platforms to enhance user adherence and engagement. Preliminary studies have already demonstrated promising outcomes using internet-based Tai Chi interventions.^[76]^ Fourth, the target populations and outcome measures of martial arts research should be expanded. While most current studies focus on older adults, future research should include adolescents and working adults. Additionally, evaluations should go beyond physical health to include psychosocial outcomes such as social participation, cultural identity, and life satisfaction. Fifth, international cooperation and the development of standardized protocols should be prioritized. Establishing globally aligned guidelines for intervention reporting and study design – such as adapting CONSORT and TIDieR frameworks for Tai Chi trials – will enhance reproducibility, transparency, and international impact.^[77]^

### 4.4. Theoretical implications and practical value

Through this bibliometric analysis of global research trends in Chinese martial arts, we not only acquired extensive objective data and identified clear developmental patterns but also gained insights into broader theoretical implications and practical value. Theoretically, this study demonstrates how traditional sports culture can undergo value reconstruction through contemporary scientific inquiry. Martial arts, originating from traditional Chinese philosophy and combat techniques, are now scientifically reinterpreted for their health-promoting functions. For instance, the health benefits of Tai Chi can be assessed and explained using Western medical indicators – such as attributing balance improvement partly to enhanced proprioception and vestibular adaptation mechanisms. This provides a vivid example of the theoretical concept of “integration of tradition and modernity,” illustrating that traditional exercise modalities are not opposed to modern science but can rather complement and enrich each other. Through scientific investigation, certain traditional concepts acquire new interpretations; for example, the concept of “Qi” can be understood in terms of psychophysiological relaxation responses. Simultaneously, traditional martial arts provide a substantial repository of intervention techniques for contemporary medicine. This paradigm of integration offers valuable insights for the scientific exploration of other traditional health practices, such as yoga and Tai Chi standing meditation (Zhan Zhuang). From an epistemological perspective, the development of martial arts research has expanded the theoretical boundaries of exercise science and rehabilitation medicine, enabling mainstream scientific systems to embrace and incorporate elements of non-Western traditional wisdom, thus achieving a more diverse and inclusive knowledge framework.

Practically, the outcomes of martial arts research have been extensively applied to clinical and community health practices, significantly benefiting the general population. For instance, Tai Chi fall-prevention programs have been widely implemented in retirement homes and community centers across numerous countries, guided by trained instructors. Research evidence has demonstrated that such programs effectively reduce fall incidence and associated healthcare costs among older adults.^[29]^ Similarly, in various cancer rehabilitation centers in the United States, Tai Chi and Qigong are employed to help patients alleviate treatment-related side effects, improve sleep quality, and enhance emotional well-being, with highly positive outcomes reported.^[43]^ Additionally, studies have explored using traditional practices like Five-Animal Exercises (Wuqinxi) to assist in drug addiction rehabilitation, yielding promising results.^[78]^ This represents an innovative expansion of martial arts applications into the domain of public health.

For China specifically, the advancement of martial arts research also holds significant value for cultural dissemination. As rigorous research is increasingly published in high-quality international journals and gains recognition among global scholars, the scientific validity and unique cultural value of Chinese martial arts are internationally acknowledged. Such recognition enhances China’s cultural soft power and facilitates the global promotion of martial arts. With the ongoing accumulation of research evidence, martial arts are expected to attract broader audiences worldwide. From a health policy perspective, governments around the world increasingly emphasize the importance of non-pharmacological interventions in chronic disease management. Martial arts research provides policymakers with valuable evidence, enabling them to confidently integrate practices such as Tai Chi into national health guidelines and fitness promotion initiatives.

In summary, the development of research on Chinese martial arts has enriched academic theory and generated far-reaching impacts on health practice. It demonstrates the modern value of traditional culture, promoting convergence among diverse cultural achievements within the medical field, and contributing positively to the global health philosophy of “prevention-oriented, mind-body harmony.” As research continues to advance, we believe Chinese martial arts will play an increasingly significant role in maintaining and promoting human health, and their scientific foundations and humanistic values will be more integrally presented to the world.

## 5. Conclusions

This study systematically mapped the global research landscape of Chinese martial arts from 1974 to 2025 using scientometric methods. The results indicate a sustained increase in scientific output, with research primarily focusing on chronic disease rehabilitation, mental health, and cognitive enhancement. A multidisciplinary research pattern has emerged, prominently represented by Tai Chi and Qigong. China and the United States remain the dominant contributors to this field; however, there remains considerable room for strengthening international collaboration. The findings underscore the growing potential of traditional martial arts as integrative mind-body interventions, offering substantial support for public health practice and cultural dissemination. Future research should prioritize mechanistic investigations, standardization of intervention protocols, and integration of intelligent technologies to build robust evidence bases. Enhanced cross-national collaboration and technological convergence will be essential for transitioning Chinese martial arts from experiential heritage to scientifically validated practices within the global health framework.

## Acknowledgments

Special thanks to my supervisor for his guidance throughout the writing of my article.

## Author contributions

**Conceptualization:** Wei Chen, Syahrul Ridhwan Morazuki.

**Data curation:** Wei Chen.

**Formal analysis:** Wei Chen.

**Funding acquisition:** Wei Chen.

**Investigation:** Wei Chen.

**Methodology:** Syahrul Ridhwan Morazuki.

**Project administration:** Wei Chen.

**Resources:** Wei Chen.

**Software:** Wei Chen.

**Supervision:** Syahrul Ridhwan Morazuki.

**Validation:** Wei Chen.

**Visualization:** Wei Chen.

**Writing – original draft:** Wei Chen, Syahrul Ridhwan Morazuki.

**Writing – review & editing:** Wei Chen.
